# Examining the Environmental Impacts of the Dairy and Baby Food Industries: Are First-Food Systems a Crucial Missing Part of the Healthy and Sustainable Food Systems Agenda Now Underway?

**DOI:** 10.3390/ijerph182312678

**Published:** 2021-12-01

**Authors:** Daniel H. Pope, Johan O. Karlsson, Phillip Baker, David McCoy

**Affiliations:** 1 Centre for Primary Care and Public Health, Queen Mary University, London E1 4NS, UK; mccoy@unu.edu; 2 Department of Energy and Technology, Swedish University of Agricultural Sciences, 756 51 Uppsala, Sweden; johan.o.karlsson@slu.se; 3 Institute for Physical Activity and Nutrition, Deakin University, Geelong 3220, Australia; phil.baker@deakin.edu.au; 4 School of Exercise and Nutrition Sciences, Deakin University, Geelong 3220, Australia

**Keywords:** breast milk, commercial milk formula, climate change, environment, water use

## Abstract

Food systems are increasingly being understood as driving various health and ecological crises and their transformation is recognised as a key opportunity for planetary health. First-food systems represent an underexplored aspect of this transformation. Despite breastfeeding representing the optimal source of infant nutrition, use of commercial milk formula (CMF) is high and growing rapidly. In this review, we examine the impact of CMF use on planetary health, considering in particular its effects on climate change, water use and pollution and the consequences of these effects for human health. Milk is the main ingredient in the production of CMF, making the role of the dairy sector a key area of attention. We find that CMF use has twice the carbon footprint of breastfeeding, while 1 kg of CMF has a blue water footprint of 699 L; CMF has a significant and harmful environmental impact. Facilitation and protection of breastfeeding represents a key part of developing sustainable first-food systems and has huge potential benefits for maternal and child health.

## 1. Introduction

Today’s food systems are driving a number of intersecting health and ecological crises that are global in scale. Food production is the leading driver of deforestation, biodiversity loss and water pollution, and generates approximately 30% of global greenhouse gas (GHG) emissions [1,2,3,4]. ‘Malnutrition in all its forms’, including unhealthy diets, undernutrition, and diet-related non-communicable diseases, are leading contributors to the global burden of disease [5,6]. Recognising these and other food-related challenges, landmark reports have recently called for urgent and transformative—some even say radical —food systems change [5,7,8,9]. According to these reports, achieving sustainable food systems—or ‘those that deliver food security and nutrition for all in such a way that the economic, social and environmental bases to generate food security and nutrition for future generations are not compromised’ [10]—must become a global political priority.

World-leading figures and organisations have taken up this call to action. The United Nations Food Systems Summit will soon ‘launch bold new actions to transform the way the world produces and consumes food’ [11]. Transforming food systems is recognised as a major opportunity for delivering on the United Nation Sustainable Development Goals (SDGs), Paris Agreement on Climate Change, and other major global development initiatives. In this paper, we argue that an important missing element from scientific reports and the global policy agenda underway is *first-foods systems*—the food systems that provide foods for infants and young children aged 0–36 months [12,13]. For example, the importance of breastfeeding has been largely ignored in recent international food systems reports, despite it representing a globally distributed and sustainable food production system—one that delivers safe, packaging-free, and individually optimised nutrition to the world’s infants and young children [14,15].

Indeed, to achieve optimal growth, development and health, the World Health Organisation (WHO) recommends infants initiate breastfeeding within the first hour of life, are exclusively breastfed for six months, and then receive nutritionally adequate and safe complementary foods while breastfeeding continues for up to two years of age or beyond [16]. This recommendation reflects long-standing evidence of the significant benefits of breastfeeding for both mother and child, and also for wider society and sustainable development. It has been estimated that achievement of the WHO’s recommendation would have prevented more than 820,000 infant deaths in 75 low- and middle-income countries in 2015, mainly due to a reduction in mortality from pneumonia, diarrhoeal illness and undernutrition [17]. Longer breastfeeding is associated with a reduced risk of obesity and type 2 diabetes [18], and increased intelligence quotient scores, which translate into increased productivity at school and work in later life [19]. Breastfeeding is also linked to beneficial effects for infants’ gut microbiota [20,21].

Maternal benefits from breastfeeding include birth spacing and a reduced risk of breast and ovarian cancers, with 27,000 deaths from breast cancer and 13,000 deaths from ovarian cancer each year preventable by universal breastfeeding [17,22]. The environmental costs of breastfeeding are negligible, while the lost economic potential from not breastfeeding is estimated at more than USD 300 billion annually [22,23]. Yet, despite this evidence, less than half of the world’s children meet the WHO’s recommendation—according to the United Nations Children’s Fund (UNICEF), only 49% of newborns initiate breastfeeding within the first hour of life, 44% are exclusively breastfed to six months, and 44% continue to breastfeed at two years of age [24].

Mirroring this slow progress is a global *infant and young child feeding (IYCF) transition* to diets higher in commercial breastmilk substitutes (BMS), defined as any milks (or products that could be used to replace milk, such as fortified soy milk) that are marketed for feeding infants and young children between the ages of 0–36 months. Commercial milk formulas (CMFs) are the main type of BMS consumed worldwide, including infant, follow-up and toddler formulas [25]. Between 2005 and 2019, global sales of CMF more than doubled, from USD 22.9 billion to USD 53.9 billion [26]. Global CMF sales per child increased 115.5% over the same period, from 3.5 to 7.5 kg/infant/year, and are projected to rise to 8.5 kg/infant/year by 2024 [12]. CMF sales are highest in Western Europe and North America, but rising most rapidly in upper-middle-income countries where sales volume per infant increased 206.9% between 2005 and 2019 [12]. Growth in East and Southeast Asia is remarkable, with compound annual growth rates in China exceeding 10% [12,23,27].

This global transition to higher milk formula diets cannot be explained by mothers and caregivers acting in isolation. Rather, it reflects wider transformations in the *first-foods systems* that structure choices and feeding practices across entire populations. A range of historical, cultural, socioeconomic and commercial factors, including the lack of maternity protection, the medicalisation of pregnancy and birthing, inadequate health service support for breastfeeding, and aggressive industry marketing and lobbying practices, all contribute to low rates of breastfeeding and help explain the global rise in CMF consumption [12,27,28,29,30,31]. The vast majority of women are physiologically able to breastfeed, with breastfeeding only rarely medically contraindicated.

Most CMF is based on cows’ milk that has been skimmed and diluted, with vegetable oils, whey protein, vitamins and minerals added to replicate some of the constituents of breast milk [15]. It is then distributed and sold in a powdered (or sometimes liquid) form, with the former requiring reconstitution with heated water and sanitised bottles for consumption. Various animal milks and other liquids (including condensed milk and plant-based milks) may also be used as functional breast milk substitutes in many (typically low-income) settings, although not marketed as such [17]. Commercial complementary foods, which often contain dairy ingredients, can further displace breastmilk, when marketed or used inappropriately [25].

Globally, CMF consumption is excessive, but it is important to distinguish between standard infant (0–6 months) CMF and follow-on (6–12 months) and toddler CMFs. Infant formula, while necessary for a small proportion of infants and mothers who are unable to breastfeed, is greatly overused, with global consumption well in excess of this background level of legitimate social need [12]. Follow-on and toddler CMF, on the other hand, is completely superfluous to human need, although demand for these products is skyrocketing, outstripping the already sizeable growth of infant CMF [27]. Furthermore, CMFs are ultra-processed food products, with toddler milks often high in added sugars, and often manifold more expensive than regular cow’s milk, despite no nutritional benefit [32,33].

While there are instances when infant CMF is clinically indicated, the general superiority of breastfeeding over feeding with milk formula for child and maternal health provides sufficient reason alone to promote and protect the choice to breastfeed [17,23,27]. However, with the threats posed by global warming and ecological degradation to population health, there is also a need to consider the environmental impact of CMF relative to breastfeeding. Indeed, several papers have recently outlined the argument that breastfeeding has a much lower environmental impact when compared to formula feeding [30,34,35,36,37].

Yet, often missing from the healthy and sustainable food systems agenda is the phenomenal global rise in CMFs and the contribution of the CMF industry to the transition of IYC diets. This paper aims to correct this neglect by contributing new information to debates about the relative environmental costs and benefits of formula feeding when compared to breastfeeding. Given that global warming and ecological degradation are arguably the defining challenge of the current era of public health, this review should also provide a useful overview for public health academics and advocates working to reduce the environmental impact of food systems more generally.

In this paper, we begin by reviewing the contribution of the dairy industry to global warming, as well as its effects on water use and pollution, air pollution, land use change, biodiversity, and soil health. We then review the evidence of the environmental impacts of CMF specifically. Finally, we consider proposed strategies to mitigate the described environmental effects of the dairy industry.

## 2. Materials and Methods

A search of the Medline and Embase databases from their origin to 22 May 2021 was conducted for peer-reviewed literature that investigated the environmental impact of commercial milk formula and the dairy industry. Journals of particular relevance (*Global Environmental Change*, *Journal of Environmental Management*, *International Journal of Greenhouse Gas Control*) and the reference lists of key articles were further reviewed for relevant articles. Titles, abstracts and full texts were assessed for relevance to the review topic. The full search strategy is presented in Appendix A.

## 3. Results

### 3.1. Dairy Industry—Greenhouse Gas Emissions

The release of greenhouse gases and the consequences for the climate have already resulted in an average global temperature rise of 1.2 °C above the pre-industrial baseline [38]. The consequences of global warming for global health are catastrophic and are already taking place. Extremes of temperature were responsible for 296,000 deaths of over 65s in 2018, an increase of more than 53% from 20 years ago, while the global land area affected by drought had doubled by 2018 compared to a historical baseline [39]. Sea level rise and wildfires threaten habitats and communities, while infectious disease transmission is projected to soar, especially vector-borne diseases. Global food security is threatened by decreasing crop and livestock yields as well as coral bleaching and sea temperature rise. Loss of livelihoods, population displacement and conflict over natural resources are further expected socioeconomic consequences [40]. Tragically, climate change widens existing health inequalities, disproportionately affecting the poorest and most vulnerable who have contributed least to the problem [39,40,41].

The food system is a major contributor to climate change, responsible for around one-third of total GHG emissions [7], with agricultural emissions expected to increase by 24% between 2012 and 2050 under a business-as-usual scenario [42]. Globally, milk is the top agricultural commodity in terms of economic value [43]. In 2019, 852 million tonnes were produced, worth more than USD 340 billion [44]. Crucially, production is forecast to increase 1.6% per annum over the next decade to 997 million tonnes in 2029 and projected to continue to increase through to 2050 [44,45]. Demand in Asia is particularly driving these increases, with compound annual growth rates of 14% in Pakistan and 12% in Vietnam and Laos [46].

Under a ‘business-as-usual’ scenario, the food system is projected to emit 1356 GtCO_2_-we cumulatively between 2020 and 2100 [47] (CO_2_ warming equivalents (CO_2_-we) calculated using GWP* is used instead of the more common GWP100 metric. This allows for aggregating short- and long-lived climate pollutants in a way relevant for long-term temperature targets [48].). This alone would far exceed the maximum allowable GHG emissions from all sources (food and non-food) for the world to retain a 50% chance of limiting global warming to 1.5 °C [47]. For the softer and, therefore more dangerous target of 2 °C, food-related emissions are on track to take up the entire annual emissions budget by 2070 [49].

Livestock is disproportionately represented in the emissions profile for the food system, contributing 57% of agricultural emissions [50]. Indeed, livestock emissions account for 14.5% of anthropogenic GHG emissions [51], having increased 51% between 1961 and 2010 [50]. In turn, ruminants are responsible for the vast majority of livestock emissions, with beef and dairy cattle responsible for 41% and 20% of livestock emissions, respectively [51]. After meat and related products, dairy products rank second in terms of food-related GHG emissions [43]. Dairy products contribute 3.1 gigatonnes of CO_2_ equivalent per year (GtCO_2_e/year), representing 3% of all anthropogenic emissions [52], and emissions from the sector are increasing [43]. Between 2005 and 2015, annual dairy-related emissions increased by 256 megatonnes of CO_2_ equivalent (MtCO_2_e), an 18% increase [53].

While carbon dioxide (CO_2_) generated by burning fossil fuels for on-farm processes, transport and electricity generation does contribute, the emissions profile of the dairy industry is dominated by methane (CH_4_) and nitrous oxide (N_2_O). Methane is generated predominantly through gastrointestinal fermentation, a fundamental aspect of ruminant digestion, and anaerobic manure decomposition. Nitrous oxide is generated by denitrification, a process in both fertilised soils and manure management [53]. It is important to distinguish between long-lived (e.g., CO_2_) and short-lived (e.g., CH_4_) greenhouse gases. Reducing CH_4_ emissions is crucial for achieving climate targets by limiting the extent of warming in the coming decades [47].

Emissions per kilogram of milk (energy-corrected for fat and protein content) vary widely due to the effect of a range of variables including location, animal genetics, farming intensity and feed types. A 2012 study of 117 farms across 38 countries including the major milk producers (EU, US, New Zealand, Brazil, India, China) estimated the global average emissions rate to be 1.5 kgCO_2_e/kg of energy-corrected milk [54]. This assessment is consistent with other similar estimates [53,55,56,57,58,59,60,61,62]. Such estimates exclude emissions generated after the farmgate (e.g., transport, processing and distribution). Including emissions from distribution to retailers and emissions due to land use change, milk is estimated to generate 3.1 kgCO_2_e/kg as a global average [63].

### 3.2. Dairy Industry—Water Pollution and Contamination

Contaminated or polluted water sources are associated with many infectious diseases including cholera, dysentery, hepatitis A, typhoid, and polio. Globally, 829,000 people die each year from poor sanitation and unsafe drinking water, including 297,000 children under the age of five years [64]. Contamination of water also decreases the productivity of fisheries as a source of food.

Dairy production has a significant effect on water quality through eutrophication, acidification, and biological and chemical pollution [65,66]. Total global livestock excreta in 2003 were estimated to contain 94 million tons of nitrogen [67]. While manure spreading contributes to nutrient recycling, in areas with high animal stocking density and little surrounding cropland where efficient spreading is challenging, nutrient loss can pollute waterways. In Europe, the livestock sector is estimated to account for 23–47% and 17–26% of nitrogen and phosphorus transported by rivers to coastal waters [68]. Excessive levels of nitrogen and phosphorus in waterways cause algal blooms, the first step in the process of eutrophication. In turn, these algal blooms reduce light penetration, with plants below dying due to lack of photosynthesis. Eventually, the algal bloom dies and decomposes, causing deoxygenation of the water and, consequently, death of larger organisms such as fish. The need to reduce eutrophication is a principal target of Sustainable Development Goal (SDG) 14 due to its adverse effects on biodiversity, food and water security [69].

Faecal bacteria contamination of water sources is another serious environmental effect of the livestock sector, with *Escherichia coli* found in concentrations up to twenty times higher in pastoral catchments than forested catchments [66]. Faecal bacteria can run off the land under rainfall and concentrate in water sources, with higher rates of campylobacteriosis, cryptosporidiosis and salmonellosis found in areas with dairy farms [70]. *E. coli* O157:H7, the major serotype causing haemolytic uraemic syndrome, has been linked to dairy farms, with instances of cattle as the source of an outbreak [71,72,73]. Chemical pollution with drug residues and heavy metals is a further cause of water contamination from dairy farms [65].

### 3.3. Dairy Industry—Other Environmental Impacts

Beyond climate change and water use and pollution, the dairy sector contributes to air pollution, zoonotic pathogens, antimicrobial resistance, land use change and biodiversity loss, and soil degradation.

Globally, more than 90% of people breathe air that exceeds WHO standards on air pollution, contributing significantly to the burden of cardiovascular and respiratory disease [74]. Annually, an estimated seven million deaths worldwide are attributable to air pollution [74]. Air pollutants (particulate matter, NOx, volatile organic compounds, ammonia) are derived from animal emissions, cropping systems, feed management, waste management, biomass burning and fossil fuel energy sources, with livestock production accounting for 8% of all PM10 (particulate matter ≤ 10 μm diameter) and 4% of all PM2.5 (≤2.5 μm diameter) emissions [75].

Demand for dairy products has resulted in 1 billion hectares, or 7% of the Earth’s land surface, being used to feed dairy animals [43]. During 2010–2014, an estimated 5.2 Mha (an area roughly the size of Costa Rica) of tropical forest was lost each year to expanding agriculture and forestry, of which roughly 3% can be attributed to global consumption of dairy products [76]. Land use change by agricultural expansion is also the single largest driver for biodiversity loss globally [77] and contributes to climate change through the loss of stored carbon and carbon sinks. 

Extensive dairy production requires large land areas and has historically been a major driver for deforestation and biodiversity loss. Intensive dairy production also affects biodiversity through deforestation and habitat loss to create arable land for feed production, as well as the introduction of new species, fertiliser use and direct effects of high cattle numbers including overgrazing. The global ruminant livestock population is enormous, numbering approximately four billion, with a total biomass that is more than ten times the total biomass of all wild mammals [78]. Specific effects of dairy production on plant and insect biodiversity are also seen. In one study of an area in New Zealand converted for intensive dairy farming, only 31% of native plant species remained after conversion and 27 new exotic species had been introduced [79]. The relationship between ecosystems, biodiversity and human health is complex but includes: ensuring adequate nutrition and food security, contributing to water and air quality, buffering of extreme weather conditions, changes to infectious disease transmission dynamics, species as sources of new medicines, and the psychological benefits of biodiverse ecosystems [65,80].

Altered land use from extensive or intensive dairy systems and the accompanying ecosystem disruption can also increase the incidence of emerging or re-emerging infectious diseases, particularly zoonotic and vector-borne diseases, mediated predominantly through changes to reservoir host populations and vector breeding sites. Zoonotic diseases are estimated to be responsible for 60% of recent emerging infectious diseases [81]. There are 45 identified zoonotic bovine pathogens, many (69%) of which are present worldwide, with a significant minority (44%) showing human-to-human transmission [82]. Antimicrobials are used frequently in the dairy industry, both prophylactically for growth promotion and in treating infections such as mastitis [83,84]. The risk of consequent antimicrobial resistance is significant [84], compounding the threat of the dairy industry increasing zoonotic and vector-borne diseases.

Intensive agriculture, including dairy farming, can degrade soil through a variety of processes: erosion, contamination, compaction, acidification, salinisation, depletion of soil organic matter, and loss of biodiversity [85]. Soil quality is essential to human health not only through its contribution to food production of high nutritional quality, but also for water filtration and prevention of airborne dust formation [86].

### 3.4. Commercial Milk Formula—Greenhouse Gas Emissions

Turning specifically to CMF, Karlsson and colleagues evaluated its contribution to climate change [35]. They assessed the carbon footprint of CMF, a commonly used methodology in which a subset of a full life cycle assessment is used to estimate the climate impact of a product, with other environmental impacts not assessed. 

The carbon footprint of production and packaging of 1 kg CMF is estimated to be between 7.1 and 11 kgCO_2_e [35]. Raw milk production is estimated to account for up to 82% of this total, with production of 1 kg of CMF requiring approximately 6.6 kg of raw milk. Adding in the emissions associated with transport, distribution, bottle production and sterilisation takes total emissions for consumption of 1 kg of CMF to between 11 and 14 kgCO_2_e. Emissions from the vitamins and minerals added to CMF could add a further 1.3 kgCO_2_e/kg CMF [35]. 

However, mothers who are breastfeeding have increased energy requirements in order to produce breast milk. This energy must be derived from food, which comes with its own carbon footprint. Based on average country-specific diets, Karlsson and colleagues estimated the carbon footprint attributable to the increased food intake of breastfeeding mothers. The additional food required to ensure healthy breastfeeding equivalent to 1 kg of CMF would have a carbon footprint of 5.9 to 7.8 kgCO_2_e. This estimate includes the emissions related to transport, distribution, food preparation and waste. 

Taken together, these results show that formula feeding has a carbon footprint that is just over double that of breastfeeding. Feeding an infant for 6 months with CMF is estimated to generate between 226 and 288 kgCO_2_e. In comparison, the increased food intake required for a mother to breastfeed for 6 months generates between 123 and 162 kgCO_2_e, an emissions profile up to 53% lower than that of CMF [35]. It is important however to note that this figure is highly sensitive to the foods that make up the mother’s diet.

### 3.5. Commercial Milk Formula—Water Use

Water use is a further area of concern [36]. Water use can be divided into three colour-coded components: green (rainwater), blue (extracted ground and surface water) and grey (water required to dilute and assimilate pollutants to meet water quality standards). 

Using a weighted global average estimate for water use in milk production [87], the 6.6 kg of raw milk used to make 1 kg of CMF [35] would require 626 L of blue water. Around 1.5 L of water is then used for every kilogram of milk processed into milk powder [88], which if used as a conservative estimate for water use in CMF processing would equate to 10 L per kg CMF. Reconstitution of CMF powder requires an estimated 7 L of water to prepare the 54 servings in 1 kg CMF [35]. 

Finally, the sterilisation of feeding bottles as per WHO recommendations requires 5 L for 6 bottles per day, totalling a further 45 L for 1 kg CMF; washing each bottle after use would amount to an additional 11 L of water for 1 kg CMF. Combining estimates for the blue water footprints of raw milk production, processing, bottle sterilisation, reconstitution and bottle washing gives a total of 699 L of water extracted from surface water or groundwater sources for 1 kg of CMF use (or 13 L of water per serving of milk formula).

However, this represents an underestimate of the true water footprint. Water used in the production of CMF ingredients other than raw milk is not included as data for these are not readily available. Green and grey water footprints further add to the overall picture, though their environmental impact is less significant than blue water use. Rainwater required in raw milk production is substantial, predominantly for feed production, and totals 6280 L for the 6.6 kg raw milk used in 1 kg of CMF [87]. Grey water use in the production of 6.6 kg of raw milk is estimated to be 524 L [87], but data for grey water use incurred in CMF processing and household consumption are not readily available.

### 3.6. Commercial Milk Formula—Other Environmental Impacts

Very little work has looked at the wider environmental impacts of CMF specifically. Given the dependence of CMF production on milk derived from the dairy industry, impacts of that sector on land use change and biodiversity loss, antimicrobial resistance, zoonoses, air pollution and soil degradation are likely to be representative of the effects of CMF.

To a greater extent than other dairy products, CMF is widely traded internationally with 1.47 million tonnes imported globally in 2019 [89]. International trade requires use of fossil fuels to facilitate its transport from production sites to consumers. Transport’s contribution to GHG emissions has been explored above, but such fossil fuel use also implicates CMF in the production of air pollutants such as particulate matter, NOx, and volatile organic compounds with deleterious effects for respiratory and cardiovascular health.

### 3.7. Proposed Mitigation Strategies

The substantial environmental effects of the dairy industry have resulted in significant attention to mitigation strategies, particularly for GHG emissions [90,91,92,93,94,95]. To date, efforts have focussed predominantly on increasing productivity through intensification and technological refinement [96]. The latter includes improving feed digestibility, type and additives to reduce enteric emissions, and changes to manure management such as acidification or fully recoupling manure recycling [97,98,99,100,101,102,103]. As a consequence, the US now produces 60% more milk with 80% fewer cows than in 1944 [104]. The concomitant GHG reductions per kilogram of milk have been significant: 43% less CH_4_ and 56% less N_2_O [104]. In the context of increasing demand, intensification can provide some climate benefit by reducing animal numbers and resource use per unit production, thereby reducing GHG emissions and the need for further land use change and deforestation [93,105].

However, even ignoring the fact that animal welfare is worsened with intensification [106], there are several reasons why intensification and technological improvements alone are not a panacea. Firstly, intensification often worsens local environmental impacts such as eutrophication and acidification, while heavy fertiliser use and monocultural pasture both negatively affect biodiversity [58,107]. Secondly, it is not fully clear that intensification will lead to an overall reduction in GHG emissions. There is, for example, disagreement on whether the larger emissions in less intensive systems are outweighed by increased soil carbon sequestration and reduced feed imports [107]. Moreover, the dairy industry does not exist within a self-contained bubble, but instead has complex relations with other industries, particularly the beef industry, with the dairy sector providing up to half of the world’s beef supply [108]. A model of GHG emissions that includes this relationship shows that increasing milk yield per cow only reduces GHG emissions if beef production also decreases. If beef production is held constant, overall GHG emissions are unchanged by dairy intensification [96,105,109].

Thirdly, the intensification efforts of the dairy sector are at risk due to climate change. An OECD-FAO report on the outlook of agriculture notes that “world production [of dairy] may be constrained because of unforeseen weather events, which affect grazing based milk production, the dominant production method worldwide” [44]. Dairy cattle are highly susceptible to heat stress due to their high metabolic rate, yet 60% of milk production is located in tropical or subtropical regions that are highly sensitive to climate change [110]. Heat stress can reduce milk output by up to 53% [111]. Furthermore, a strong negative correlation between heat tolerance and milk production traits has been observed in dairy cattle [112]. Adaptation to climate change is, therefore, likely to reverse some of the climate mitigations achieved by intensification.

The argument of Jevon’s paradox is also relevant: intensification drives down costs, potentially increasing output and/or affordability and thereby increasing demand. The increased output to meet this demand overcompensates for any reduction in emissions, reversing the environmental benefits of intensification.

Finally, and perhaps most importantly, even without the negative effects of climate change on dairy production, intensification and technological strategies appear to be insufficient to meet emissions reductions targets [49,56,113,114,115,116,117,118]. Maximal ‘technical mitigation’ of the global food system could reduce food system emissions in 2050 from 12 GtCO_2_e/year to 8.3 GtCO_2_e/year, still far above the target of 5 GtCO_2_e/year by 2050 that would allow a 66% probability of limiting global warming to 2 °C [7,49].

Although successful climate change mitigation depends on fewer cattle, a limited number of ruminant animals can play a role, for example by converting non-human edible biomass to food, which is important in organic mixed-farming systems [119,120]. Karlsson and colleagues also showed that reducing soybean imports to the EU to reduce deforestation pressure would favour ruminants over pigs and poultry [121]. Introduction of perennial forage crops into crop rotations can also improve soil health and reduce input requirements [122].

Nonetheless, reducing demand for dairy products is deemed an essential step in mitigating the environmental impacts of the dairy industry [7,49,56,90,116,117,123]. CMF, a dairy-based product that is inappropriately and excessively used and which has a readily available environmentally sustainable alternative that is cheaper, and nearly always better and safer for health, represents an area of demand that could and should be significantly reduced.

## 4. Discussion

Food systems are coming to be understood as an increasingly important public health issue [7,47,56]. Provision of nutrition through the production, distribution and consumption of food has a clear and direct effect on health, while indirect effects of food systems on health are broad and include social and economic consequences, antimicrobial resistance, and environmental effects.

In this paper, we consider a small part of the global food system: the ‘boom’ in milk formula consumption [12]. Of particular concern is the fact that a healthier, cheaper and more environmentally friendly source of nutrition is being replaced with an inferior, more expensive and more environmentally damaging product for millions of infants and young children.

The drivers for this ‘global transition’ in infant and young child nutrition have been explored elsewhere [12,23,27,28] but include: a lack of awareness of the benefits of breastfeeding, the power of marketing including violations of the WHO’s International Code of Marketing of Breastmilk Substitutes, work-related pressures and a lack of maternity rights protections, and beliefs that manufactured products are generally better than natural products [23,27,28,29,30,31,124,125,126].

The ‘boom’ in milk formula consumption is also aided by the fact that its environmental impacts are not fully accounted for in the market price of CMF, representing an externalisation of costs that fall onto all people, including future generations, and disproportionately affecting poorer and marginalised populations who are most vulnerable to the effects of global warming and ecological breakdown [30]. These environmental costs and the consequences for health add to existing arguments for the promotion and protection of breastfeeding on health grounds [17,22,23].

Previous work has established the environmental benefits of breastfeeding over formula feeding, with the carbon footprint of formula use up to two times that of breastfeeding [35]. Water use, driven primarily by the use of milk as a main ingredient, has also received attention [36]. Even using conservative estimates, we find that use of 1 kg of milk formula requires 699 L of ‘blue’ water to be extracted from surface or ground water sources and consumes well in excess of 6000 L of ‘green’ rainwater.

**Figure 1:**
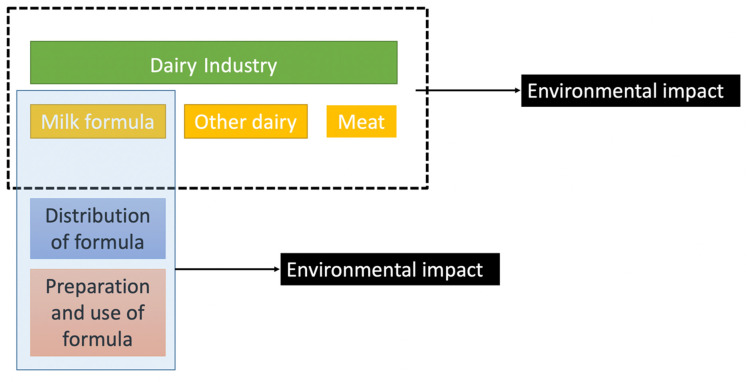
A framework for understanding the environmental impacts of formula use and the dairy industry and their interconnections.

Food systems are complex and interlinked, making isolated product assessments challenging. The environmental impact of formula is dominated by its use of milk as an ingredient, with raw milk production accounting for 82% of formula’s carbon footprint [35]. This dependency of formula on the dairy industry means that an understanding of the environmental impact of formula requires an understanding of the impacts of the dairy sector. A framework for understanding the interconnections of formula and dairy and their environmental effects is presented in Figure 1.

Livestock are disproportionately over-represented in the emissions profile of the food system, with dairy products contributing 3.1 GtCO_2_e/year, or 3% of all anthropogenic emissions [52]. Production of milk also has a significant water footprint. Driven principally by use for feed production, an average of 1125 L of water are required to produce 1 kg of milk [87]. The dairy sector contributes to water pollution and contamination, accounting for a significant portion of nitrogen and phosphate loading into waterways [68]. Water pollution and contamination also affects the clean water supplies needed for the consumption of CMF itself. Finally, air pollution, land use, biodiversity loss, soil degradation, and effects on infectious disease transmission and antimicrobial resistance are further detrimental impacts of the dairy sector on which the CMF industry is heavily reliant. The social and economic role of the dairy industry is beyond the remit of this review.

While the dairy industry has used intensification and technological improvements to reduce GHG emissions per kg of milk produced, intensification may worsen local environmental effects [58,107] and is ultimately insufficient on its own to achieve safe emissions levels. Ultimately, reductions in dairy production and consumption are needed to stay within our planetary boundaries [49,56,113,114,115,116,117,118]. Given the ready availability of a healthier and more environmentally sustainable alternative (breastfeeding), CMF represents an area of dairy demand that should be significantly reduced.

When considering CMF within the global food system, it is important to note that while it mostly displaces breastfeeding, some formula is consumed in place of other foodstuffs. Follow-on formulas are increasingly used in infant diets in lieu of locally available foods [12,27]. The growth and expansion of the market for CMF may act not only as a gateway for even greater levels of consumption of ultra-processed foods by children (with negative health consequences), but may, in some circumstances, be displacing cheaper and less environmentally damaging alternative foods. Diversion of expenditure from nutritious foods, education or healthcare to CMF can comprise significant portions of household budgets in lower-middle-income countries and represents a significant opportunity cost for individuals and wider society. Moreover, families diverting their income to purchase CMF also spend more on medical care than those who do not buy CMF [127].

While there are important and legitimate uses of CMF, current use is far above levels that are appropriate or desirable. The industrial solution of developing plant-based rather than dairy-based CMF may bring some environmental benefit, but its inferiority compared to breastfeeding for maternal and child health means facilitating breastfeeding should be the primary goal of the global health community. Reducing CMF consumption to sustainable levels and facilitating breastfeeding requires a whole-of-systems approach. This includes a strong role for government intervention through policy and regulation. Implementation of the WHO International Code of Marketing of Breastmilk Substitutes is central to these efforts.

## 5. Conclusions

In the context of unfolding climate and ecological crises, healthcare professionals and public health advocates have a responsibility to better understand the environmental impacts of food systems, including first food systems, and champion sustainable transitions.

The environmental impact of commercial milk formula is significant and represents a clear argument for promoting and protecting breastfeeding. The dairy industry has a sizeable and harmful environmental impact, contributing significantly to the environmental impact of breast milk substitutes. The consequences of these environmental effects for public health are serious and reducing demand for dairy is deemed essential to stay within planetary boundaries. Advocating for such a transition in our food system represents an important role of the global public health community.

## Data Availability

Not applicable.

## Appendix A

The following search strategy was used for the PubMed database:

((milk) OR (milk formula) OR (dairy)) OR (breast milk substitut*)) AND ((climat*) OR (greenhouse ga*) OR (warming) OR (emissio*) OR (CO_2_) OR (CH_4_) OR (N_2_O) OR (carbon dioxide) OR (methane) OR (nitrous oxide) OR (water) OR (eutrophication) OR (nitrogen) OR (phosphate) OR (pollution) OR (biodiversity))

Keywords from this strategy were used to search journals of particular relevance: *Global Environmental Change*, *Journal of Environmental Management*, *International Journal of Greenhouse Gas Control*.

Titles and abstracts were assessed for relevance to the review topic: the environmental impact of commercial milk formula and the dairy industry. Full texts of relevant articles were reviewed. Reference lists of key articles were reviewed for further relevant references.
